# Did you choose appropriate mouthwash for managing chemoradiotherapy-induced oral mucositis? The therapeutic effect compared by a Bayesian network meta-analysis.

**DOI:** 10.3389/froh.2022.977830

**Published:** 2023-01-30

**Authors:** Xue Wang, Li Zeng, Xue Feng, Na Zhao, Na Feng, Xin Du

**Affiliations:** Medical Center of Hematology, The Second Affiliated Hospital, Army Medical University, Chongqing, China

**Keywords:** oral mucositis, cancer, radiotherapy, chemotherapy, mouthwash, network analysis

## Abstract

**Background:**

Oral mucositis (OM) is one of the most common adverse effects of radiotherapy and chemotherapy. It greatly affects the patients' quality of life and hinders cancer treatment implementation. Treating OM with mouthwash is a widely used strategy that can effectively relieve symptoms and promote healing. However, the wide mouthwash selection confuses clinicians. This Bayesian network meta-analysis aimed to compare the effects of various mouthwash types used to treat OM and provide high-level evidence-based recommendations for OM treatment.

**Methods:**

Database search included PubMed, Embase, Cochrane Library, and Web of Science from inception to April 21, 2022. The primary outcome was OM score improvement following the World Health Organization grades. The randomized controlled trial (RCT) bias risk assessment tool provided in the Cochrane Handbook assessed the studies' risk of bias. We performed pairwise and Bayesian network meta-analysis with random effects following the PRISMA guideline.

**Results:**

The study included 13 RCTs with 570 patients. Pairwise comparisons showed that povidone-iodine was more effective than chlorhexidine (weighted mean difference [WMD], −2.64; 95% confidence interval [CI], −2.72 to −2.56) but inferior to granulocyte-macrophage colony-stimulating factor (GM-CSF; WMD, 0.20; 95% CI, 0.06–0.34) after one week of mouthwash treatment. Vitamin E (WMD, −0.94; 95% CI, −1.03 to −0.85), natural drugs (WMD, −0.93; 95% CI, −1.46 to −0.40), and phenytoin (WMD, −0.38; 95% CI, −0.59 to −0.17) exhibited better therapeutic effects than a placebo after three weeks of treatment. Bayesian network meta-analysis showed that povidone-iodine was superior to chlorhexidine in treating OM (WMD, 2.63; 95% CI, 0.20–5.01). Other mouthwashes showed no significant differences. Rank probability indicated that the best OM therapeutic mouthwashes were GM-CSF (54%), vitamin E (24%), and natural drugs (43%) after one, two, and three weeks of treatment, respectively.

**Conclusion:**

GM-CSF was the most effective mouthwash type for OM treatment. When considering the cost and effectiveness, povidone-iodine and sodium bicarbonate might be the most advantageous. Furthermore, natural drugs have the same potential in treating OM. Safety and acceptability are their most outstanding characteristic.

## Introduction

Cancer is one of the leading cause of human death (1). According to authoritative data from the International Agency for Research on Cancer (IARC), about 19.29 million patients were newly diagnosed with cancer worldwide, and 9.96 million died due to cancer in 2020, posing a serious threat to human health (2). Radiotherapy and/or chemotherapy are the main treatments for cancer in the clinic, effectively improving survival and prognosis. However, their accompanying adverse effects greatly affect the patients’ quality of life and hinder the implementation of treatment plans (3).

Oral mucositis (OM) is one of the most common complications caused by radiotherapy and chemotherapy. Almost all patients receiving head and neck radiotherapy, and 20%–40% of those receiving chemotherapy develop OM (4–6). Among the underlying mechanisms, the antineoplastic drugs and radiation can directly interfere with the renewal and metabolism of normal epithelial cells, induce epithelial cell apoptosis, and disturb the oral microenvironment, leading to oral mucosa injury, secondary inflammation, and the development of ulcerative lesions (7, 8).

Patients receiving chemotherapy could experience some oral discomfort within 5–10 days, while patients receiving radiotherapy develop OM within 1–2 weeks after treatment (9). The most prominent clinical symptoms of OM are ulcerative pain and the resulting difficulty in swallowing and eating (10). The World Health Organization (WHO) categorized OM into four grades according to its symptom. Patients with Grades 1 and 2 can tolerate eating, while about 70% of those with Grades 3–4 switch to tube feeding because of severe pain. Some patients might develop depression, which greatly reduces their quality of life, increases the hospital stay and related costs, and even leads to treatment interruption in severe cases (11, 12). Therefore, OM treatment for patients undergoing radiotherapy and chemotherapy is of great significance for improving their prognosis.

**Figure 1:**
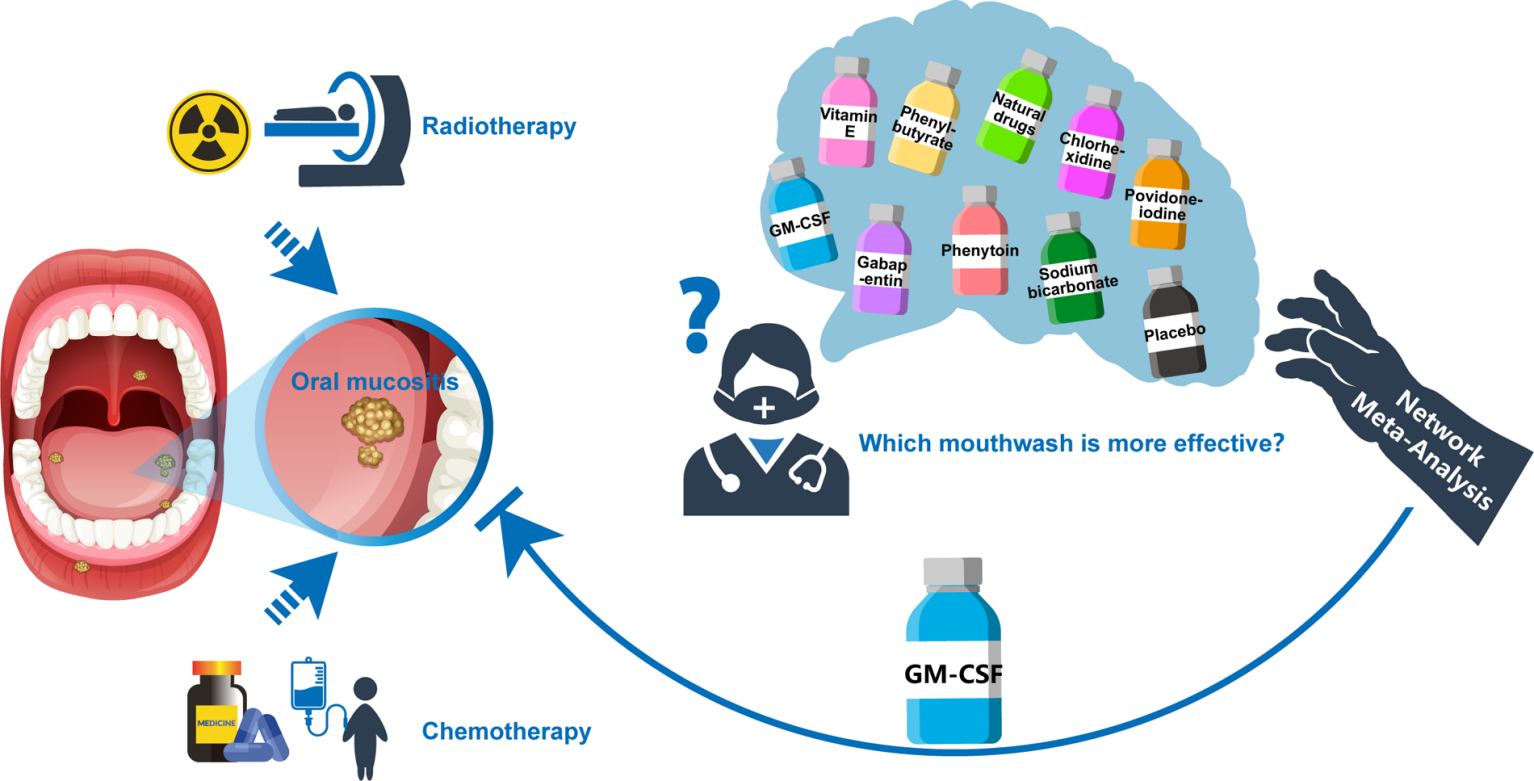
The summarised diagram of this Bayesian network meta-analysis. Abbreviations: GM-CSF, granulocyte-macrophage colony-stimulating factor.

During OM treatment, mouthwash application could keep the oral environment clean and with fewer bacteria, thereby lowing the risk of infection following mucosal damage (13, 14). More importantly, mouthwash can effectively relieve OM symptoms, reduce OM grade, and promote OM recovery. However, clinically applied mouthwashes have diverse compositions, and a unified induction and comparison between them is still lacking, seriously hindering the optimization and selection of OM treatment. Although a related meta-analysis compared the preventive effect of several types of mouthwash on OM (13), the reference value is still relatively limited, especially for treating already present OM. This study performed a Bayesian network meta-analysis of the existing literature reporting OM treatment with mouthwashes. The specific effects of various mouthwash types in treating OM were compared, providing a theoretical and reference basis for clinical treatment of OM caused by radiotherapy and/or chemotherapy (Figure 1).

## Materials and methods

### Evidence acquisition

This Bayesian network meta-analysis followed the Preferred Reporting Items for Systematic Reviews and Meta-Analyses (PRISMA) guideline (15). Since the data analyzed in this Bayesian network meta-analysis were derived from previously published studies, no procedure on human participants was performed by any of the authors.

### Search strategy

We searched four commonly used electronic databases, PubMed, Embase, Cochrane Library, and Web of Science, from their inception to April 21, 2022, without restricting the regions or publication type but restricting the language to English. The searched used the following combined search terms: (oral mucositis OR stomatitis OR dental ulcer OR mouth ulcer OR oral ulcer OR canker sore) AND (mouthwash OR mouth rinse OR mouth wash) AND (radiotherapy OR radiation treatment OR radiation therapy OR chemotherapy OR chemical therapy OR chemical treatment). The reference lists of the included studies and reviews were searched manually to identify reports not returned by the electronic search.

### Inclusion and exclusion criteria

We included studies that assessed the clinical efficacy of various mouthwashes vs. placebo or other agents in radiation/chemotherapy-induced OM. The predetermined study inclusion criteria were: 1) study design: randomized controlled trial (RCT); 2) population: patients with OM caused by radiotherapy/chemotherapy; 3) intervention: two or more mouthwash types; 4) outcome: OM severity score reported based on the WHO grading system; 5) duration of follow-up: 7–28 days. We excluded studies comparing the incidence of OM following prophylactic use of mouthwash, intervention studies with insufficient valid data (such as no statistical OM severity score displayed) that could be extracted, and retrospective studies, conference papers and abstracts, reviews, letters, and editorials.

### Data extraction and risk of bias assessment

Two independent investigators (WX and LZ) assessed all candidate studies based on their titles and abstracts and eliminated duplicates and studies that did not meet the inclusion criteria. Disagreements during the data extraction process were decided by the adjudicating senior authors (NZ, NF and XD). A standardized Bayesian network meta-analysis tool was used to extract data from the full texts of the included studies: author, publication year, region, study design, type of therapy (chemotherapy, radiotherapy), type of mouthwash, sample size, patient baseline characteristics (sex, age), primary outcomes, and follow-up time. Subsequently, we used the RCT bias risk assessment tool of Cochrane Handbook 5.3.0 (https://training.cochrane.org/handbook) to assess the risk of bias in the included RCTs from the following seven aspects: random sequence generation, allocation concealment, blinding of participants and personnel, blinding of outcome assessment, incomplete outcome data, selective reporting, and other biases. The assessment results were scored as low, high, and unknown risk.

### Outcome measures

The primary outcome measure for this network analysis was the OM score improvement (post-treatment score minus pre-treatment score) using the WHO grades. The included studies were subdivided into 1-, 2-, and 3-week comparison subgroups based on the follow-up observations. It should be noted that we selected primary outcome indicators as close to the subgroup time as possible for studies in which the observation time differed from the subgroup cut-off point (for example, the results of day 10 were classified into the 1-week comparison group). Animal and plant extract mouthwashes were grouped as natural drug mouthwashes.

### Data synthesis and statistical analysis

We pooled the results of all the direct and indirect comparisons to assess the OM score improvements in the various types of mouthwash. The results are reported as weighted mean differences (WMDs) with the corresponding 95% confidence intervals (CIs).

We used Stata, Version 12.0 (Stata Corporation, College Station, TX) to build a network for comparing the included mouthwashes, visually reflecting the direct and indirect relationship among them by a geometric pattern. Subsequently, we conducted pair-wise meta-analyses with a random-effects model to synthesize studies and obtain pooled evidence for direct comparisons. Statistical heterogeneity across studies was assessed by forest plots and the inconsistency statistic (*I*^2^). Statistical significance was set as *P* < 0.05.

We then built a random-effects network within a Bayesian framework using the Markov chain Monte Carlo methods in ADDIS, Version 1.15 (Aggregate Data Drug Information System, https://addis.drugis.org). We set the variance scaling factor to 2.5 to fit the model, used four chains for simulation, set the number of simulation iterations to 100,000 (the first 20,000 iterations were used for tuning the iterations to remove the effect of the initial value, and the subsequent 80,000 iterations were used for sampling), and set the thinning interval to 10. All indirect comparative evidences of OM score improvement (WMD) available by this network with *P* < 0.05% and 95% CI that did not include zero were considered statistically significant. Furthermore, we calculated the ranking probabilities of the various mouthwash types by calculating the WMD for each when compared with an arbitrary common control group and counting the proportion of iterations of the Markov chain in which each mouthwash had the highest WMD, the second-highest WMD, and so on.

Lastly, we evaluated the inconsistency within the Bayesian network meta-analysis by calculating the variance with ADDIS. A significant inconsistency was concluded if the random effects standard deviation between the consistency and inconsistency models was large or the inconsistency factor was not close to zero. In such cases, we adjusted the study inclusion according to a quantitative estimation, ultimately obtaining an ideal consistent network.

## Results

### Eligible studies

**Figure 2:**
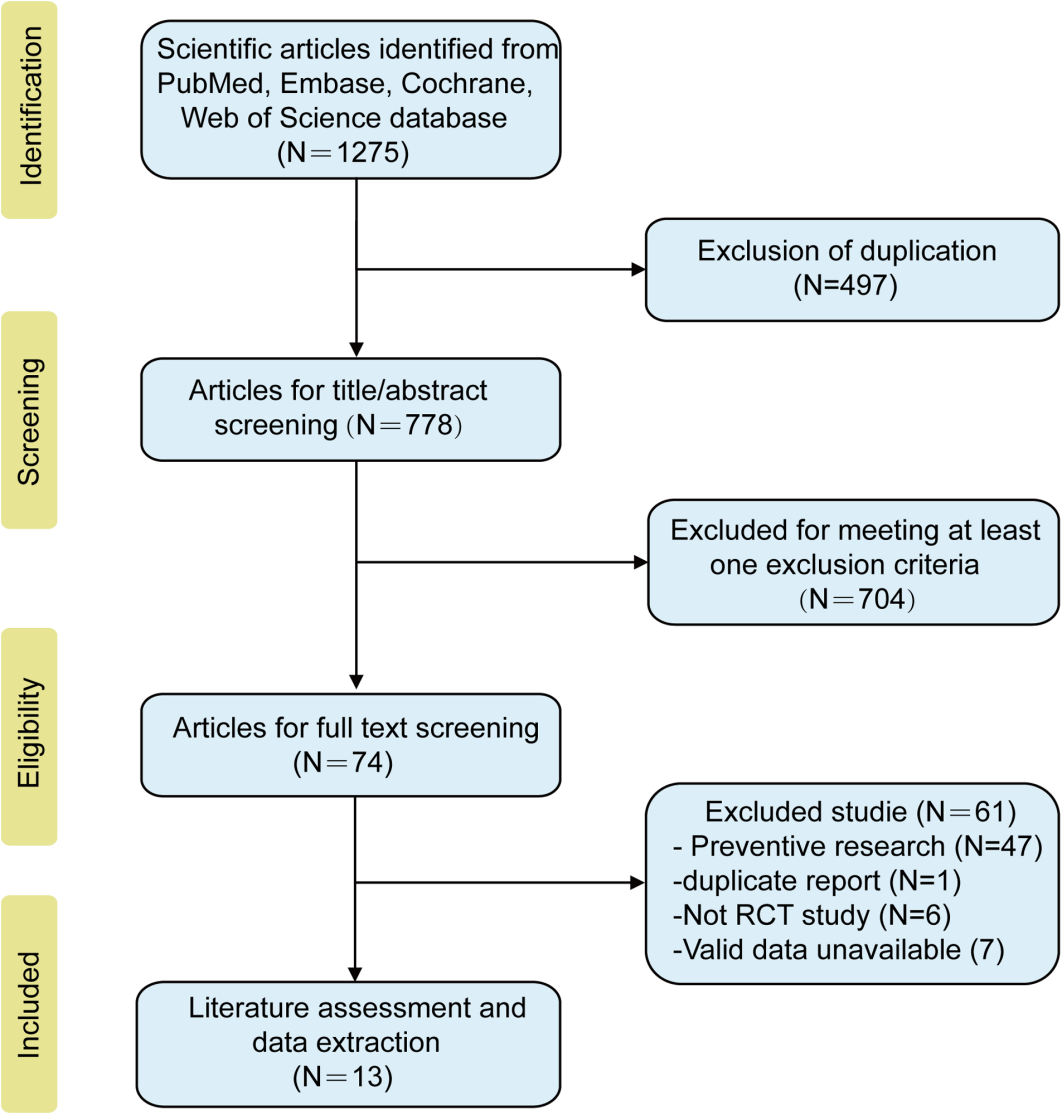
PRISM flow diagram of the study selection for this Bayesian network meta-analysis.

The initial search yielded 1,275 publications, of which 497 duplicate records were removed. The titles and abstracts of the remaining 778 publications were reviewed, and 704 were excluded for reporting on unrelated topics or not reporting data of interest (such as no statistical OM severity score displayed). After screening the full text of the remaining 74 studies, we excluded 47 that had a prevention design, one duplicate report, six that were not RCT, and seven without valid data. Finally, 13 studies met the inclusion criteria and were included in this Bayesian network meta-analysis (16–28). The literature search and study selection procedure is presented in Figure 2.

### Characteristics of eligible studies

**Figure 3:**
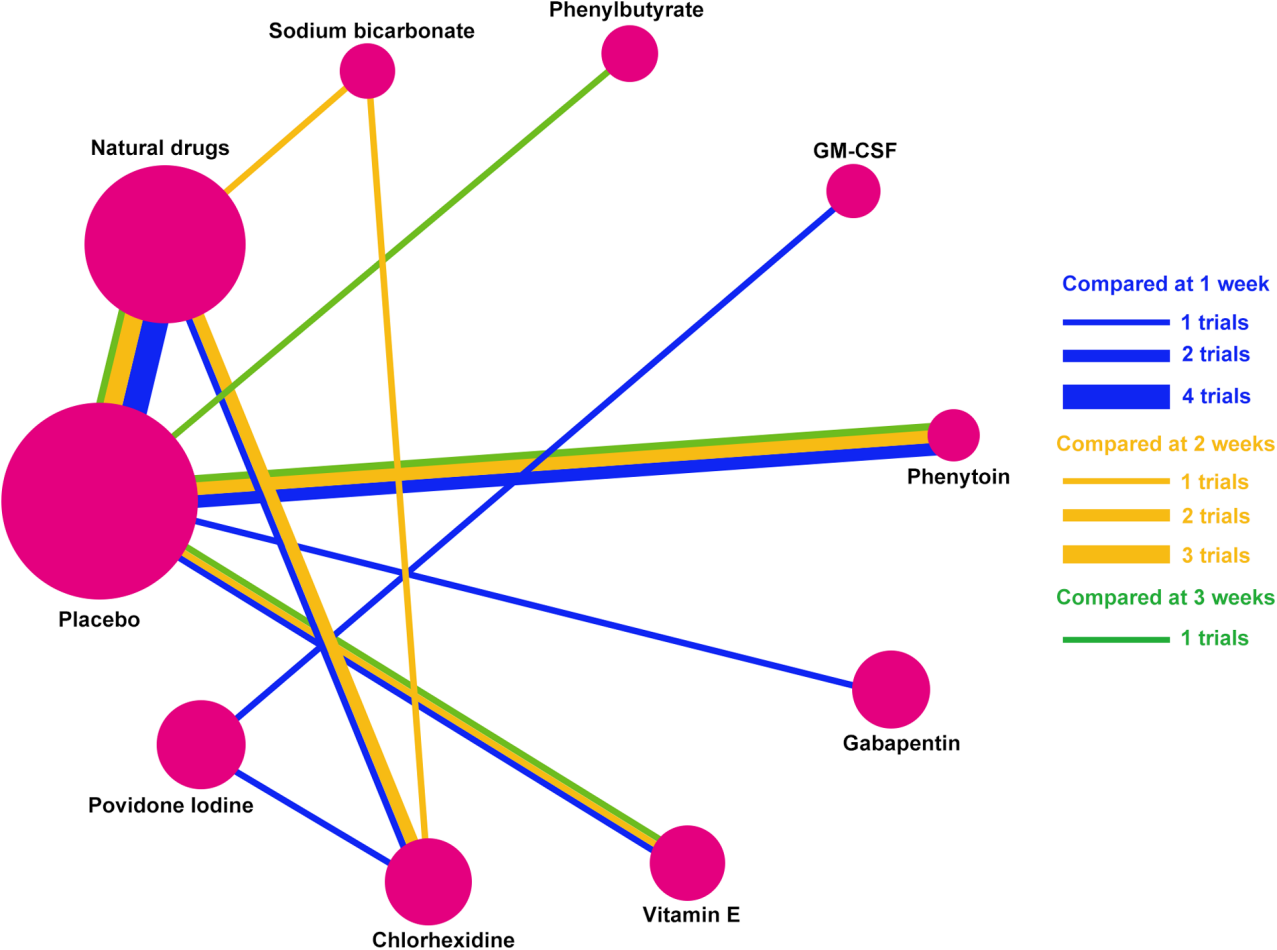
Network established for multiple mouthwash comparisons. Solid lines between mouthwashes represent direct comparisons. The circle area represents the sample size. Abbreviations: GM-CSF, granulocyte-macrophage colony-stimulating factor.

**Figure 4:**
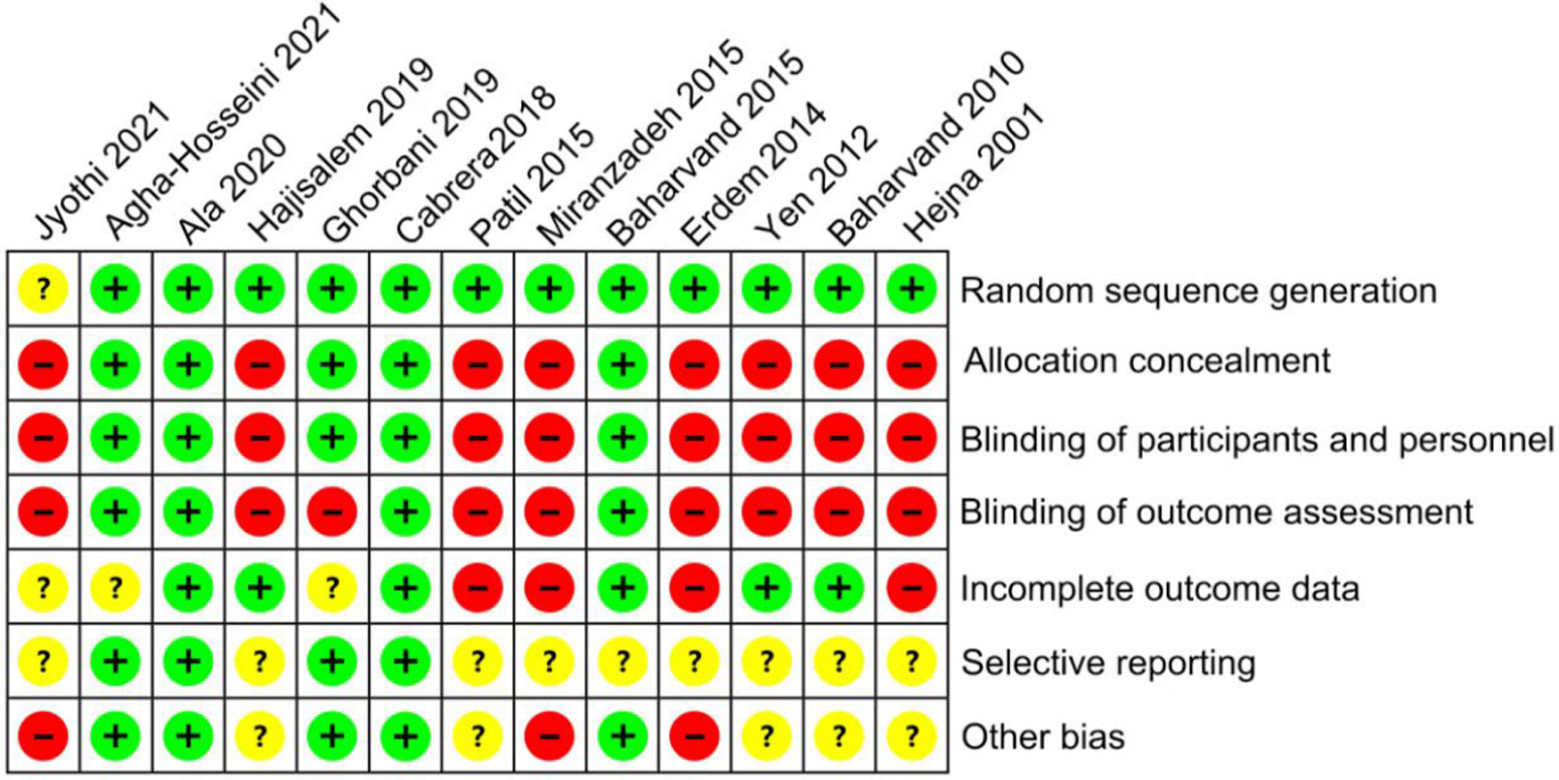
Risk of bias assessment of the 13 RCTs included in this Bayesian network meta-analysis.

Ten mouthwash types were investigated in the 13 eligible studies, including povidone-iodine, chlorhexidine, vitamin E, gabapentin, phenytoin, GM-CSF, phenylbutyrate, sodium bicarbonate, natural drugs (including Achillea extract, Althaea root extract, Plantago major extract, Curcumin, and Royal jelly), and placebo. The comparative relationships between the mouthwashes are shown in Figure 3. These studies were published between 2001 and 2021, covering a variety of tumor types treated by radiotherapy and/or chemotherapy. Among them, one study reported radiotherapy-induced OM (17), seven reported chemotherapy-induced OM (18–21, 23, 27, 28), and five reported chemoradiotherapy-induced OM (16, 22, 24–26). Seven of these studies were performed in Iran (17–20, 23, 24, 27), two in India (16, 22), and one each in Spain (21), Turkey (25), China (Taiwan) (26), and Australia (28). The total number of patients included in this Bayesian network meta-analysis was 570. The sample size varied across studies, ranging between 12 and 103. The enrollment OM grade (Grades 1–4) and post-treatment follow-up (3–28 days) also varied among studies. The main characteristics of the included studies are summarized in Table 1, and the study risk of bias assessment results are shown in Figure 4.

**Table 1:** Baseline characteristics of the trials included in this Bayesian network meta-analysis.

Study	Year	Country	Cancer type	Treatment	OM scores	Mouthwash	Patients (Male/Female)	Age (mean ± SD)	Follow-up assessments
Jyothi et al. (16)	2021	India	Multi type	RT/CT	Grades 1–4	Povidone Iodine	37/13	25–65	Days 3, 5, 7
						Chlorhexidine			
Agha-Hosseini et al. (17)	2021	Iran	Head and neck cancer	RT	Grades 3–4	Vitamin E	17/12	55.03 ± 9.84	Weeks 1, 2, 3, 4
						Placebo	17/13	55.57 ± 11.53	
Ala et al. (18)	2020	Iran	Multi type	CT	Grades 1–4	Gabapentin	9/22	58.67	Days 10
						Placebo	14/13	56.84	
Hajisalem et al. (19)	2019	Iran	Acute myeloid leukemia	CT	Grades 1–4	Natural drug (Achillea extract)	5/9	44.71 ± 16.60	Days 5, 10, 15, 20
						Placebo	7/8	41.86 ± 12.50	
Ghorbani et al. (20)	2019	Iran	Multi type	CT	Grades 1–3	Natural drug (Althaea root extract)	11/14	53.96 ± 15.48	Days 7, 14
						Placebo	11/14	49.48 ± 16.80	
Cabrera-Jaime et al. (21)	2018	Spain	Multi type	CT	Grades 2–3	Sodium bicarbonate	24/26	59.5 ± 14.3	Days 5, 7, 14
						Natural drug (Plantago major extract)			
						Chlorhexidine			
Patil et al. (22)	2015	India	Head and neck cancer	RT + CT	Grades ≥1	Natural drug (Curcumin)	5/5	60	Days 10, 20
						Chlorhexidine	6/4	59	
Miranzadeh et al. (23)	2015	Iran	Multi type	CT	Grades 1–4	Natural drug (Achillea extract)	12/16	56.46 ± 14.32	Days 7, 14
						Placebo	12/16	55.54 ± 14.01	
Baharvand et al. (24)	2015	Iran	Head and neck cancer	RT/+CT	Grades 1–3	Phenytoin	1/7	52.75 ± 13.23	Days 7, 14, 21
						Placebo	5/3	56.00 ± 14.65	
Erdem et al. (25)	2014	Turkey	Multi type	RT + CT	Grades 1–3	Natural drug (Royal jelly)	20/31	50.69 ± 25.42	Days 7, 14
						Placebo	28/24	53.03 ± 13.08	
Yen et al. (26)	2012	China (Taiwan)	Head and neck cancer	RT + CT	Grade 1	Phenylbutyrate	11/6	51.1 ± 10.6	Days 28
						Placebo	17/2	54.8 ± 12.1	
Baharvand et al. (27)	2010	Iran	Multi type	CT	Grades 2–3	Phenytoin	3/3	38.8 ± 13.8	Days 7, 14
						Placebo	2/4	33.3 ± 14.8	
Hejna et al. (28)	2001	Austria	Multi type	CT	Grades 1–3	GM-CSF	5/10	58	Days 3, 6
						Povidone Iodine	9/7	73	

CT, chemotherapy; GM-CSF, granulocyte-macrophage colony-stimulating factor; RT, radiotherapy; OM, oral mucositis; SD, standard deviation.

### Direct meta-analysis

**Figure 5:**
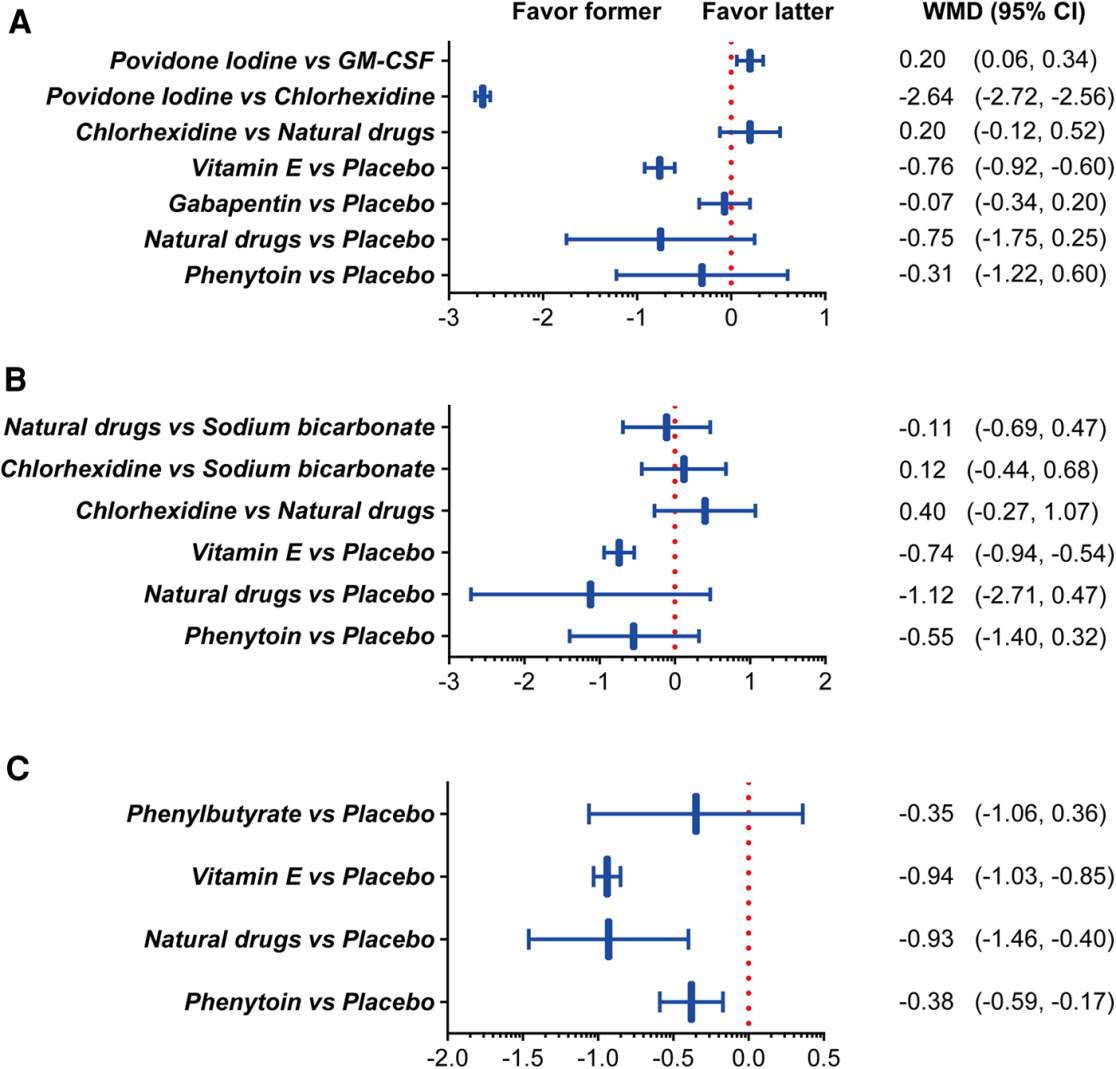
Combined forest plots showing comparisons between the mouthwash types based on their OM score improvement after one (**A**), two (**B**), and three (**C**) weeks of treatment. Abbreviations: CI, confidence interval; GM-CSF, granulocyte-macrophage colony-stimulating factor; WMD, weighted mean difference. Note: Natural drugs in Figure **A** include Achillea extract, Althaea root extract, Plantago major extract, Curcumin, and Royal jelly; in Figure **B** include Achillea extract, Althaea root extract, Plantago major extract, and Royal jelly; in Figure **C** include Achillea and Curcumin.

Figure 5 shows the results of direct pairwise comparisons between the mouthwashes. One week after treatment, vitamin E displayed a better therapeutic effect than placebo (WMD, −0.76; 95% CI, −0.92 to −0.60). Povidone-iodine was more effective than chlorhexidine (WMD, −2.64; 95% CI, −2.72 to −2.56), but less effective than GM-CSF (WMD, 0.20; 95% CI, 0.06–0.34). Two weeks after treatment, vitamin E remained superior to the placebo (WMD, −0.74; 95% CI, −0.94 to −0.54), while the other mouthwashes showed similar effectiveness. Vitamin E (WMD, −0.94; 95% CI, −1.03 to −0.85), natural drugs (WMD, −0.93; 95% CI, −1.46 to −0.40), and phenytoin (WMD, −0.38; 95% CI, −0.59 to −0.17) exhibited superior therapeutic effect to the placebo after three weeks of treatment.

### Network meta-analyses

**Figure 6:**
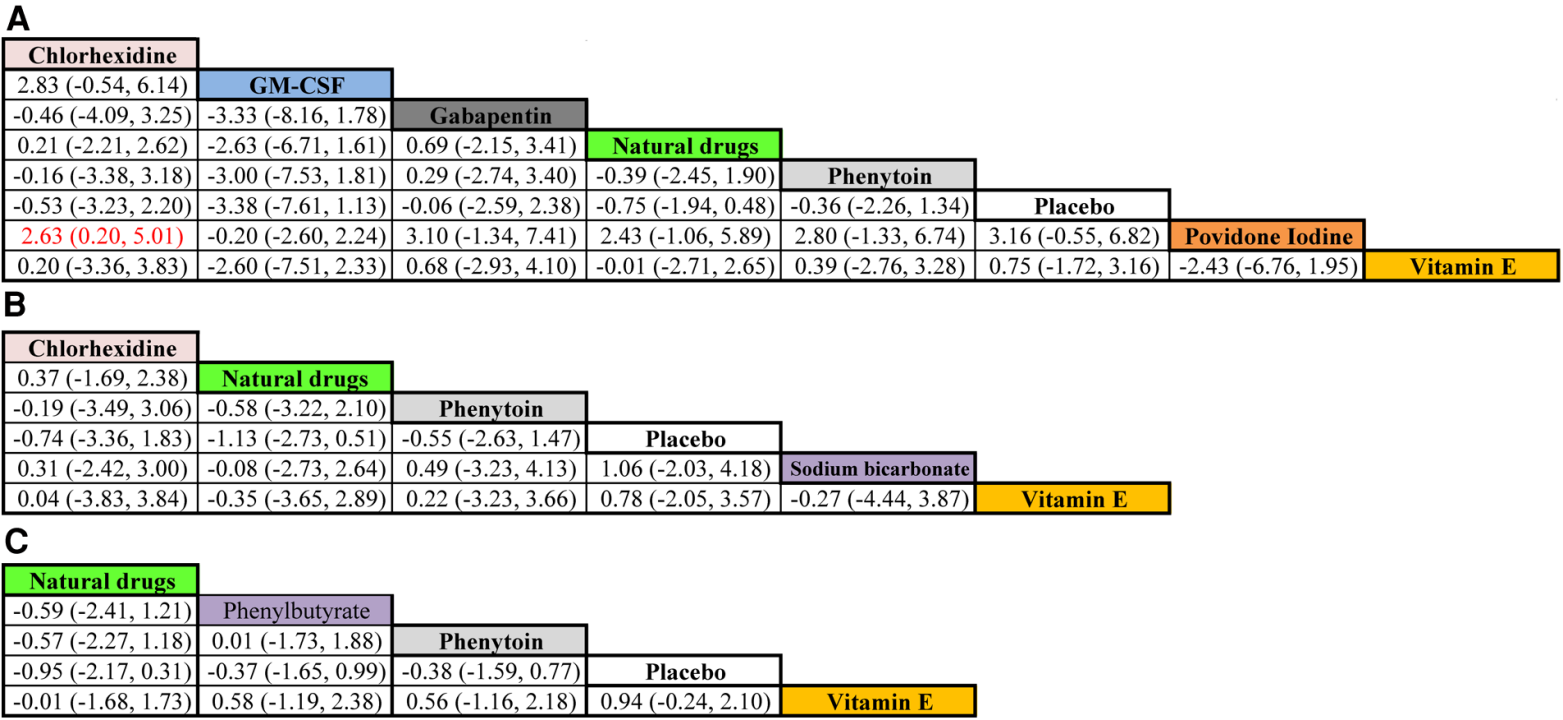
Comparative efficacy of the mouthwashes based on their OM score improvement after treatment for one (**A**), two (**B**), and three (**C**) weeks. Cells present WMD (95% credible interval) for comparisons between column- and row-defining mouthwashes. Red cells indicate significant differences. WMD > 0 favors the row-defining mouthwash, and WMD < 0 favors the column-defining mouthwash. Abbreviations: GM-CSF, granulocyte-macrophage colony-stimulating factor; WMD, weighted mean difference. Note: Natural drugs in Figure **A** include Achillea extract, Althaea root extract, Plantago major extract, Curcumin, and Royal jelly; in Figure **B** include Achillea extract, Althaea root extract, Plantago major extract, and Royal jelly; in Figure **C** include Achillea and Curcumin.

The therapeutic effects of the ten mouthwash types on OM score improvement are shown in Figure 6. Eight mouthwash types evaluated the effect on OM score improvement after one week of treatment (chlorhexidine, GM-CSF, gabapentin, natural drugs, phenytoin, placebo, povidone-iodine, and vitamin E). Among these, povidone-iodine was superior to chlorhexidine (WMD, 2.63; 95% CI, 0.20–5.01) in improving OM score. The mouthwashes evaluated after two weeks of treatment were chlorhexidine, natural drugs, phenytoin, placebo, sodium bicarbonate, and vitamin E, and those evaluated after three weeks of treatment were natural drugs, phenylbutyrate, phenytoin, placebo, and Vitamin E. However, the WSD of these mouthwashes were similar after two and three weeks of treatment (95% CI contained 0 for all).

### Rank probability

**Figure 7:**
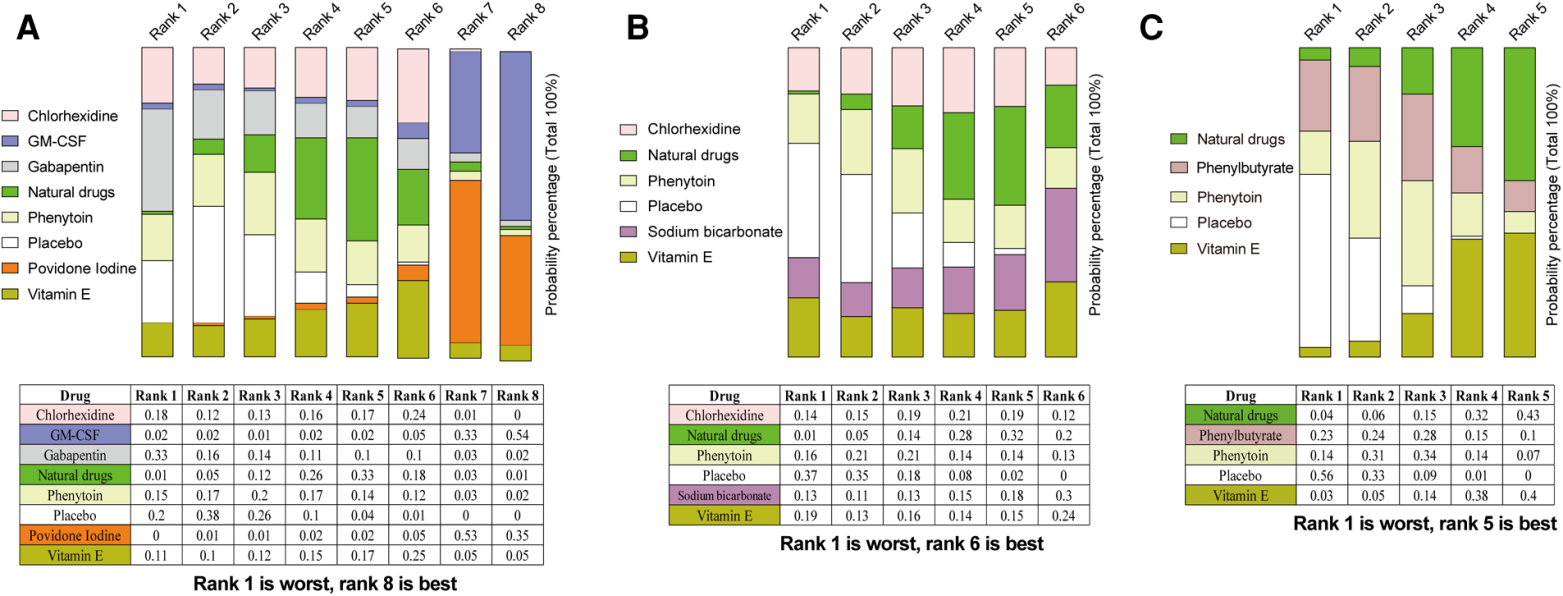
Ranking probabilities based on multiple comparisons for OM score improvement after treatment for one (**A**), two (**B**), and three (**C**) weeks. The total probability of a single histogram is 100%, and a larger band indicates a higher probability in this ranking. The worst effect was Rank 1 and the best, Rank 8. Abbreviations: GM-CSF, granulocyte-macrophage colony-stimulating factor. Note: Natural drugs in Figure **A** include Achillea extract, Althaea root extract, Plantago major extract, Curcumin, and Royal jelly; in Figure **B** include Achillea extract, Althaea root extract, Plantago major extract, and Royal jelly; in Figure **C** include Achillea and Curcumin.

We performed probability ranking for the OM score improvement after mouthwash treatment to obtain more informative results for the ten mouthwash types (Figure 7). After one week of treatment, GM-CSF had the best therapeutic effect, followed by povidone-iodine, vitamin E, natural drugs, chlorhexidine, phenytoin, placebo, and gabapentin. After two weeks of treatment, sodium bicarbonate performed best, followed by natural drugs, while the placebo showed the worst performance. The placebo displayed the worst performance after three weeks of treatment. Unlike in the 2-week assessment, natural drugs were the best mouthwash type after three weeks of treatment, followed by vitamin E.

## Discussion

OM is one of the most common adverse events following cancer radiotherapy and chemotherapy, yet no specific medicine is currently available (6). Mouthwash is a practical and well-accepted OM treatment strategy that can effectively relieve the patients' symptoms and promote healing. However, the wide variety of mouthwashes confuses clinicians when considering OM treatment options. Therefore, obtaining high-level evidence-based data and comparing the therapeutic effects the various mouthwash types have on OM could help solve this dilemma. In this respect, our study is of fundamental clinical significance.

This study investigated the comparative therapeutic effect of ten mouthwash types on chemotherapy and/or radiotherapy-induced OM using a Bayesian network meta-analysis. Our results showed significant differences in the efficacy among mouthwashes at time points after treatment. For example, GM-CSF had the highest OM score improvement after one week of treatment, suggesting it is the best choice for treating OM. GM-CSF is a central cytokine that regulates myeloid hematopoiesis in the bone marrow (29). It can selectively act on myeloid hematopoietic stem cells, promote their proliferation and differentiation, increase undifferentiated granulocyte function, and enhance the chemotactic ability of macrophages and neutrophils (30, 31). Therefore, GM-CSF could improve the phagocytic activity and bactericidal ability of anti-inflammatory cells, help keep the oral microenvironment relatively clean and inhibit the infection-leaded inflammatory response, ultimately promoting ulceration healing (32). However, just because of the function of GM-CSF on myeloid hematopoietic stem cells, its application in leukemia patients needs careful consideration (especially in myeloid leukemia). As the most used external disinfectant in clinical practice, povidone-iodine could also treat OM. The patients' symptoms are relatively mild at the early stages of the disease, and the degree of personal attention is relatively low (33). In such cases, using the expensive GM-CSF mouthwash is likely to result in poor patient compliance, while povidone-iodine is just right because of its low price. It can address the clinical needs of such patients making it a good treatment option for some patients with mild symptoms.

Sodium bicarbonate had the best therapeutic effect after two weeks of treatment, possibly due to its excellent ability to dissolve bacterial mucin. Sodium bicarbonate could effectively alleviate the toxicity caused by oral acidic metabolites, inhibit fungi growth, protect the ulcer surface, reduce saliva viscosity, and neutralize oral acidity, promoting OM healing (34). It should be noted that we could not conclude which mouthwash was best among sodium bicarbonate, GM-CSF, and povidone-iodine after two weeks of treatment because comparative data on GM-CSF and povidone-iodine were lacking. This aspect needs further assessment in future clinical studies. Additionally, we found that natural drugs had a time-dependent characteristic in treating OM; they had relatively limited efficacy in the early treatment stages, but their efficacy gradually improved with the prolongation of treatment, becoming most effective after three weeks of treatment. Considering their good biological safety and high patient acceptance, we believe that natural drugs' advantages in treating OM are mainly reflected in their long-lasting efficacy and safety. They are suitable for patients with mild OM and a long course of chemoradiotherapy or as a complementary treatment to other mouthwashes.

In addition to screening for the best OM treatments, this study helped detect several ineffective mouthwash types, some even with adverse effects. The efficacy of gabapentin was similar to that of the placebo after one week of treatment, and it had a lower ranking probability than the placebo. Gabapentin is a central nervous system drug with antiepileptic, anticonvulsant, and analgesic effects (18). Using gabapentin mouthwash to treat OM can theoretically relieve the pain caused by OM. However, the clinical outcomes are not ideal. In fact, the pharmacological effects of gabapentin mainly depend on the inhibitory neurotransmitter *γ*-aminobutyric acid in the central nervous system (35); local or external application of gabapentin cannot fully exert its function. Furthermore, many studies have shown that gabapentin had specific cytotoxic effects on the liver, kidney, and hematopoietic system (36–38), making it likely to aggravate the damage to the mucosal epithelial cells when used locally, especially when the oral-mucosal barrier dysfunctions.

This Bayesian network meta-analysis had several limitations. First, although all included studies were RCTs, their sample sizes were relatively small, and the study quality varied, possibly increasing the risk of bias. Second, the various cancer type, different chemotherapy and radiotherapy protocols may lead to heterogeneity between studies, but current number of included studies is not sufficient for subgroup analysis. Last, the mutual comparisons between nodes in the Bayesian network constructed in this study were not comprehensive enough, being limited by the number of published studies. Fortunately, the conclusions of the consistency and inconsistency models in this study were highly consistent, suggesting that the Bayesian network meta-analysis had good repeatability and credibility.

## Conclusion

This was the first Bayesian network meta-analysis study investigating the therapeutic effect of various mouthwash types in treating chemoradiotherapy-induced OM. After comparing and analyzing ten mouthwash types (povidone-iodine, chlorhexidine, vitamin E, gabapentin, phenytoin, GM-CSF, phenylbutyrate, sodium bicarbonate, natural drugs, and placebo), we consider GM-CSF the most effective mouthwash for OM treatment. When considering the cost and effectiveness, povidone-iodine and sodium bicarbonate might be more advantageous. Besides, natural drugs had the same potential in treating OM. Safety and acceptability are their most outstanding characteristics. With the continuous deepening of related research, we believe that new mouthwash types will be discovered and applied, increasing the diversity of treatment options for OM.

## Data Availability

The raw data supporting the conclusions of this article will be made available by the authors, without undue reservation.
